# A Rare Case of Primary Peritoneal Carcinoma With Large Bowel Metastasis

**DOI:** 10.7759/cureus.34167

**Published:** 2023-01-24

**Authors:** Aishwarya Jangam, Vanitha Math

**Affiliations:** 1 General Surgery, Logan Hospital, Brisbane, AUS; 2 Obstetrics and Gynaecology, Gold Coast University Hospital, Gold Coast, AUS

**Keywords:** case presentation, presentation, rare cancer, epithelial ovarian cancer, metastasis to the colon, primary peritoneal carcinoma

## Abstract

Primary serous peritoneal carcinoma (PPC) is a rare malignancy often presenting with a significant disease burden and a poor prognosis. A 65-year-old female was seen in the surgical outpatient clinic with a two-month history of weight loss, altered bowel habits and a CT scan characterizing a heterogenous right paracolic gutter mass and suspicious liver lesions. At colonoscopy, a prominent appendiceal orifice was biopsied to be poorly differentiated carcinoma favouring gynaecological tract origin. The patient was admitted with an acute small bowel obstruction secondary to progression of metastases and underwent neoadjuvant chemotherapy with a near complete response on repeat staging. A debulking hysterectomy, bilateral salpingo-oophorectomy, pelvic peritonectomy and small bowel nodule excision were performed. The diagnosis of PPC was confirmed when no malignancy was found in the pelvic organs. The presence of intraluminal colonic metastasis with PPC is exceedingly rare with this being only the third such case in the literature.

## Introduction

Primary peritoneal carcinoma (PPC) is a rare epithelial cancer closely associated histologically and in clinical behavior with epithelial ovarian carcinoma. As such they are classified and treated together as one clinical entity by the International Federation of Gynaecology and Obstetrics [1]. High-grade serous carcinoma is the most common subtype of epithelial ovarian, fallopian tube and peritoneal carcinomas, accounting for 70-80% of cases, and is considered fundamentally different from low-grade serous carcinomas due to its molecular features [2]. TP53 and BRCA mutations are gene mutations typically present in PPC with the immunohistochemistry featuring P53+, WT1+, Pax8+ and High Ki67. Age is a common risk factor with median age of diagnosis being 60-65 years. The literature quotes a 2% risk increase for each additional year under the age of 50 years and an 11% increase for each year above 50 years of age. Other risk factors include early menarche, late menopause, family history, BRCA variants, Lynch syndrome, nulliparity, smoking, asbestos exposure and previous history of pelvic irradiation [2].

Unfortunately, high-grade serous carcinoma carries a poor overall prognosis and due to its aggressive nature, most patients present in a subacute fashion with 75% of patients presenting with Stage III or Stage IV disease [2,3]. Acute presentations of PPC include malignant ascites, malignant pleural effusion, small bowel obstruction and venous thromboembolism [4]. Fifty percent of patients have distant metastases at diagnosis and twenty percent present with regional lymph nodes [2]. The presence of intraluminal bowel metastasis from PPC is a very rare occurrence and has been reported only twice in the literature [5,6].

## Case presentation

**Figure 1:**
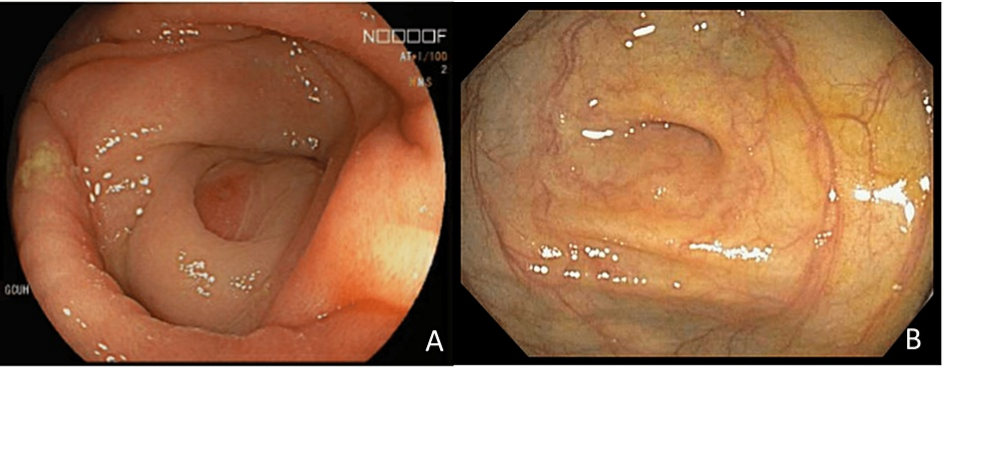
Panel A shows the prominent appendiceal orifice seen at colonoscopy. Panel B depicts a normal appendiceal orifice.

**Figure 2:**
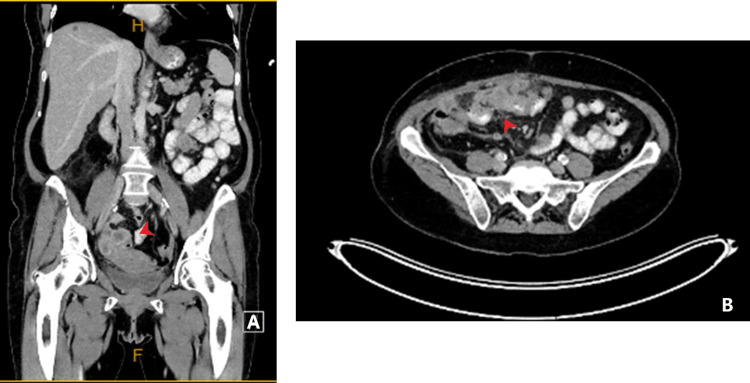
CT scan demonstrating progressive peritoneal disease and a developing small bowel obstruction. Panel A demonstrates the close association of the peritoneal disease with the right ovary and panel B shows the developing small bowel obstruction.

A 65-year-old female was seen in the surgical outpatient clinic with two months of weight loss, altered bowel habits and bloating. Her medical history included hyperlipidemia, hypertension and a previous appendicectomy. She had early menarche, was nulliparous and an active smoker. She did not have any history of hormonal therapy or a family history of malignancy. The only pertinent physical examination finding was mild generalized abdominal tenderness. Computed tomography (CT) scan of the abdomen showed a heterogenous mesenteric mass in the right paracolic gutter with associated lymphadenopathy and multiple suspicious parenchymal liver lesions. The patient had an elevated CA125 level of 245U/mL and a transvaginal ultrasound was normal. At colonoscopy, a slightly prominent appendiceal orifice was biopsied (Figure 1), the histology of which returned as poorly differentiated carcinoma favouring a gynaecological tract origin with immunohistochemistry positive for WT1 and PAX8 [7]. Two weeks after the colonoscopy, the patient was admitted with acute small bowel obstruction. Repeat CT imaging showed that this was secondary to progression of peritoneal disease with new involvement the right ovary (Figure 2) leading to the provisional impression of a primary right ovarian carcinoma. The obstructive symptoms improved with non-operative management.

**Figure 3:**
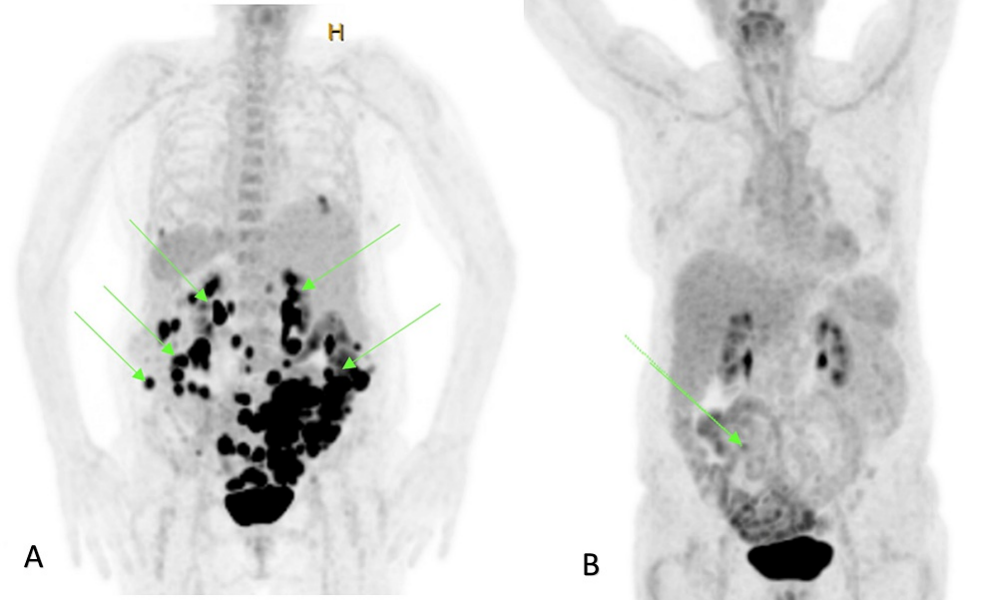
PET/CT before (A) and after (B) neoadjuvant chemotherapy showing near complete response. Arrows indicate PET-avid lesions.

After multidisciplinary team consultation, the patient received inpatient neoadjuvant chemotherapy with six cycles of carboplatin/pacilitaxel to near complete response. A repeat fluorodeoxyglucose (FDG)-positron emission tomography and CT scan (FDG PET/CT) showed only two areas of residual omental deposits (<15mm) compared to the pre-chemotherapy imaging (Figure 3). She underwent laparotomy, hysterectomy, bilateral salpingo-oophorectomy, pelvic peritonectomy, omentectomy and a small bowel nodule excision to a macroscopically R0 resection. The intraoperative findings noted the small bowel serosal nodule as the only abnormality and the pelvic organs were normal. The diagnosis of PPC was confirmed when histology showed normal gynaecological structures, and the small bowel serosal nodule was the only tissue with remnant carcinoma. The patient had an uncomplicated recovery and underwent adjuvant platinum-plus-taxane chemotherapy. Unfortunately, the patient presented two months post-chemotherapy completion with a painful abdominal wall nodule at the superior aspect of the midline laparotomy wound. This was initially thought to be an infected seroma but further imaging and subsequent biopsy confirmed this as a metastatic recurrence communicating with the peritoneal cavity. She is currently planned to undergo palliative radiotherapy.

## Discussion

This case is remarkable for both the rare occurrence of an intraluminal bowel metastasis secondary to PPC and for its demonstration of the varied presentations and aggressive clinical behaviour of PPC. Intraluminal bowel metastasis of PPC is exceedingly rare. The pathway of dissemination is thought to be via the transcoelomic route - spreading through the peritoneum [8]. Often patients with bowel metastasis can present with symptoms similar to primary bowel cancer - as seen in this case [8]. Interestingly, the caecum in this patient appeared normal at the staging laparoscopy. Given the rarity of bowel metastasis there is little literature addressing whether visible changes are seen post-chemotherapy. Furthermore, the macroscopic impression of the appendiceal orifice prominence on colonoscopy was documented as unlikely to be a malignancy or a mucocele. The histological findings in this case reiterate the importance of approaching any abnormalities with a high degree of suspicion in patients with peritoneal carcinomatosis, particularly if they carry risk factors - in this patient they were age over 65 years, smoking, nulliparity and early menarche.

The preferred treatment for Stage III and IV epithelial ovarian cancers including PPC is primary surgical cytoreduction followed by systemic chemotherapy - ideally a platinum-plus-taxane combination. The aim of surgical treatment is to achieve <10mm of residual disease macroscopically. Neoadjuvant chemotherapy is offered to patients with unresectable disease, patients unlikely to be optimally cytoreduced if taken to theatre at the time of presentation or those who are poor initial operative candidates [2,9]. Those unlikely to tolerate surgery at any point can be offered chemotherapy alone with the aim of tumour control. Furthermore, as exemplified by this case, even with good response the risk of recurrence in this aggressive malignancy is 62% for all stages and 80-85% for women who presented with Stage III or IV disease with further treatment guided by signs and symptoms [8,10].

## Conclusions

PPC is a rare and aggressive cancer and should be considered as a differential in patients with peritoneal disease and relevant risk factors, particularly to allow for counselling regarding the associated poor prognosis. In addition to the unusual presence of a large bowel metastasis, the rapid progression of symptomatology in this case demonstrates both the subacute and acute presentation of PPC and the appropriate change in management adapting to the changing clinical scenario as per the guidelines and literature.
